# Cardiovascular Drugs and Osteoarthritis: Effects of Targeting Ion Channels

**DOI:** 10.3390/cells10102572

**Published:** 2021-09-28

**Authors:** Raminta Vaiciuleviciute, Daiva Bironaite, Ilona Uzieliene, Ali Mobasheri, Eiva Bernotiene

**Affiliations:** 1 Department of Regenerative Medicine, State Research Institute Centre for Innovative Medicine, LT-08406 Vilnius, Lithuania; raminta.vaiciuleviciute@imcentras.lt (R.V.); daiva.bironaite@imcentras.lt (D.B.); ilona.uzieliene@imcentras.lt (I.U.); ali.mobasheri@imcentras.lt (A.M.); 2 Research Unit of Medical Imaging, Physics and Technology, Faculty of Medicine, University of Oulu, FI-90014 Oulu, Finland; 3 Departments of Orthopedics, Rheumatology and Clinical Immunology, University Medical Center Utrecht, 508 GA Utrecht, The Netherlands; 4 Department of Joint Surgery, First Affiliated Hospital of Sun Yat-sen University, Guangzhou 510080, China

**Keywords:** osteoarthritis, cardiovascular drugs, hypertension, vascular dysfunction, cartilage, chondrocyte, ion channels

## Abstract

Osteoarthritis (OA) and cardiovascular diseases (CVD) share many similar features, including similar risk factors and molecular mechanisms. A great number of cardiovascular drugs act via different ion channels and change ion balance, thus modulating cell metabolism, osmotic responses, turnover of cartilage extracellular matrix and inflammation. These drugs are consumed by patients with CVD for many years; however, information about their effects on the joint tissues has not been fully clarified. Nevertheless, it is becoming increasingly likely that different cardiovascular drugs may have an impact on articular tissues in OA. Here, we discuss the potential effects of direct and indirect ion channel modulating drugs, including inhibitors of voltage gated calcium and sodium channels, hyperpolarization-activated cyclic nucleotide-gated channels, β-adrenoreceptor inhibitors and angiotensin-aldosterone system affecting drugs. The aim of this review was to summarize the information about activities of cardiovascular drugs on cartilage and subchondral bone and to discuss their possible consequences on the progression of OA, focusing on the modulation of ion channels in chondrocytes and other joint cells, pain control and regulation of inflammation. The implication of cardiovascular drug consumption in aetiopathogenesis of OA should be considered when prescribing ion channel modulators, particularly in long-term therapy protocols.

## 1. Introduction

Osteoarthritis (OA) is the most common form of arthritis, affecting approximately 7% of the global population, which is more than 500 million people worldwide, with people over 50 and women disproportionately affected [1,2]. OA is a whole joint disease, and since the synovial joint is regarded as an organ, many constituent tissues are affected, including articular cartilage [3], subchondral bone [4,5,6], synovium [7,8,9], infrapatellar fat pad [10,11], meniscus [12] and intraarticular ligaments [13]. However, the articular cartilage component has received the most attention due to the fact that its degradation and loss can be assessed by radiography using X-rays and magnetic resonance imaging (MRI) [14,15]. Newer compositional MRI and ultrasound methods can detect synovial inflammation, damage to intraarticular ligaments and infrapatellar fat pad volume and give deeper insights into the initiation, progression and eventual treatment of the disease [16,17,18,19].

OA can be classified into multiple phenotypes based on molecular mechanisms or clinical biomarkers—inflammatory, metabolic syndrome, mechanical overload, chronic pain, bone and cartilage metabolism phenotype, etc.—which are mechanistically linked together [20]. The most common OA symptoms are pain, stiffness, swelling, deformity and reduced function of joints [21]. EULAR and ACR OA treatment guidelines mainly involve pharmacological, non-pharmacological and surgical modalities. Pharmacological treatment includes pain and inflammation management by oral and topical non-steroidal anti-inflammatory drugs (NSAIDs), opioid drugs and corticosteroid interventions, while glucosamine, chondroitin sulphate and hyaluronic acid showed no direct evidence to support clinical benefits. Non-pharmacological treatment recommends weight loss or local muscle strengthening. Knee or hip joint replacement is used for those for whom other treatment modalities have failed [22,23]. However, neither effective treatment for OA [24] nor a means for its early diagnosis or evaluation of drug efficacy is currently available [25]. Moreover, the precise regulation of signalling pathways of chondrogenesis remains unclear [26], and the regulation of cartilage extracellular matrix (ECM) turnover is challenging.

Chondrocytes comprise the only cells in cartilage tissue, and they are responsible for the production of ECM: collagen II, glycoproteins and proteoglycans [27,28]. The ionic and osmotic environment of cartilage has a profound impact on the cellular physiology of chondrocytes, including the expression and function of ion channels and the turnover of ECM [29,30]. Recently, the dependence of chondrocyte functions on the homeostasis of intracellular ions such as calcium, potassium and sodium has been shown [31]. Chondrocytes are non-excitable cells, but voltage-gated ion channels regulate resting membrane potential and protect cells from osmotic shock [32]. Chondrocytes have a whole range of ion channels to enable ion transport and electrical signalling across the plasma membrane, the collection of which is referred to as chondrocyte ‘channelome’; however, the regulation and maintenance of intracellular ion balance in the cartilage is a complicated and poorly understood process [32,33]. The proper influx and/or release of the intra-/extracellular ions is crucial for the metabolic functioning of chondrocytes [34,35]. In addition, the chondrocyte channelome is known to be involved in the regulation of mechanotransduction, chemotransduction and osmoregulation in the cartilage [36]. It has been shown that altered levels of receptors and ion channels in the human synovial fluid progenitor cell membrane can be potential biomarkers of OA [37]. Therefore, one of the ways of potentially preventing OA disorder can be the regulation of ion channels.

Cardiovascular diseases (CVD) are a group of heart and blood vessel disorders, the treatment of which mainly involves drugs modulating various ion channels [38,39,40]. According to the European Society of Cardiology, CVD includes coronary heart disease, heart failure, dilated cardiomyopathies, arrhythmias, aortic and peripheral arterial diseases, hypertension and more. The main groups of CVD medications are β-adrenoblockers, blockers of the angiotensin–aldosterone system, nitrates (nitroglycerine and its derivatives), calcium channel blockers and antiarrhythmic drugs. All these drugs, in one way or another, can influence other tissues in the body, including cartilage [41]. CVD is a well-known age-related health care problem worldwide, which, as a rule, mainly affects the elderly population with overweight and metabolic disorders who also have a high prevalence of OA. Furthermore, OA is suggested as a risk factor for CVD, which, given the high prevalence and incidence of OA and CVD in the general population, has high clinical and public health importance [42]. Data related to the effects of CVD drugs on cartilage, chondrocytes and other joint tissues and cells in the various stages of OA development and disease progression are lacking.

The aim of this review is to discuss the potential effects of currently used CVD drugs on cartilage, synovium and subchondral bone, focusing on their mechanisms of action on ion channels in joint cells, pain control, regulation of inflammation and possible consequences on the progression of OA. Drugs developed to treat CVD may have indirect impacts on the synovial joint, affecting chondrocyte function, with either beneficial or harmful consequences. Therefore, it is important to determine if some CVD drugs may be adjusted for the treatment of specific OA phenotypes in patients with CVD. In this review, we summarized current information related to the two chronic diseases, CVD and OA, their origin, causes and inter-relationships at the molecular level, drug consumption and pharmacological treatment. We have also reviewed the mechanisms of action of the ion channel modulating CVD drugs and their possible implication in the cartilage ECM turnover, subchondral bone remodelling and the course of OA. The understanding of the complex action of CVD drugs could help to solve important therapeutic issues, how to avoid their adverse effects and whether pharmacological treatment for CVD can be beneficial for patients with the metabolic type of OA.

## 2. The Association between OA and CVD

Arrhythmia, hypertension and cardiac ischemia are the most prevalent diseases, particularly in elderly and obese individuals with limited physical activity and metabolic disorders, causing low-grade inflammatory conditions [43]. A CANTOS study showed the IL-1β role in CVD together with the effect on OA course. It proved the inflammatory relation between OA and CVD, showing that the anti-inflammatory medicine canakinumab reduced cardiovascular event rates together with incidence rates of total hip or total knee replacement [44]. Hypertension, along with hypercholesterolemia, hypertriglyceridemia, hyperglycaemia and increased waist fat are components of the metabolic syndrome, which is one of the main risk factors for OA with a metabolic disease phenotype [45]. It was shown that limitation of physical activities due to knee and hip OA significantly worsens the condition of the cardiovascular system, while after joint replacement, fewer cardiovascular events are experienced [46,47]. There is also emerging evidence that CVD is a co-morbidity factor for many OA patients [48,49].

### 2.1. Risk Factors for OA and CVD

**Figure 1:**
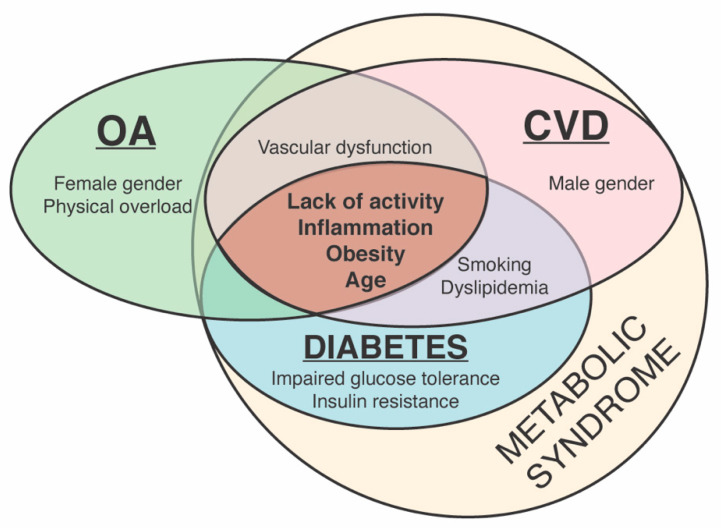
Common and distinct risk factors for osteoarthritis (OA), cardiovascular diseases (CVD) and diabetes and their relation to metabolic syndrome.

Although the aetiopathogeneses of OA and CVD are different, they share several similar risk factors. Preventable risk factors, including reduced physical activity, obesity, vascular dysfunction and inflammation, are closely related not only to OA and CVD but also diabetes, and the symptoms of these diseases, such as elevated blood pressure and blood glucose and a high level of waist fat, are constituents of metabolic syndrome (Figure 1) [50,51,52].

#### 2.1.1. Common Risk Factors for OA and CVD

OA is a patient age-related joint disease and most often diagnosed at ages above 40 years old [53]. Different studies show different averages of OA patients’ ages, ranging between 52 and 62 years old [54]. CVD is also associated with age: the number of new cases is highest in the population older than 65 years [55]. Rational physical exercise was shown to increase the content of glycosaminoglycans (GAGs) and decrease the risk of OA [56,57]. In vitro studies showed the positive effect of mechanical loading to chondrocytes. Murine chondrocytes, bovine chondrocytes and cartilage explants were associated with reduced inflammatory IL-1β signalling chondrocytes after cyclic mechanical loading [58]. Osteochondral cylinders isolated from bovine stifle joints showed upregulated COL2A1 and ACAN gene expression and increased metabolic activity after high load [59].

Modest physical activity is also beneficial in CVD by decreasing hypertriglyceridemia [60,61]. Obesity is a significant risk factor for both CVD and knee OA with slightly different mechanisms [62]. At first, obesity increases weight and causes mechanical overload of the load-bearing joints, e.g., knee joints, causing direct damage to cartilage [63]. Another effect is the increase in mediators of low-grade inflammatory conditions, i.e., IL-6, both in the joint tissue and cardiovascular system in obese patients [64,65]. Vascular dysfunction is directly associated with hypertension and heart failure, although subchondral bone ischemia leads to cartilage damage [66,67]. There is also an interaction between metabolic syndrome-related disorders, such as insulin resistance, abdominal obesity, hypertension and hyperlipidaemia, and morbidity with OA or CVD [68,69,70].

#### 2.1.2. Distinct Risk Factors for CVD and OA

Impaired vascular endothelial functions are known to be the main cause of atherosclerosis and hypertension [71,72]. Cartilage does not have the vascular system [73]; however, the systemic endothelial dysfunction also affects joint tissue. Higher levels of endothelin-1 (ET-1) are associated with atherosclerosis [74], while there is also evidence concerning the ET-1-induced enzymatic destruction of articular cartilage [75]. ET-1 stimulates vascular endothelial growth factor (VEGF) secretion, which increases atherosclerotic plaque formation and is an early marker of cartilage and subchondral bone degeneration [76,77].

Women are more likely to develop OA than men, especially after age 50, whereas CVD is more typical for males [78,79]. Smoking is associated with an increased risk of hypertension and myocardial infarction; however, so far, there has been a controversial association with OA [80,81]. A few reports even suggest that current smokers have a lower risk of OA [82,83,84]. The protective mechanism is not clear; however, it is known that smokers usually have a lower BMI [82]. Nicotine was shown to increase ECM synthesis and enhance chondrocyte metabolism in a rat model [85]. Other studies confronted these findings, concluding that there is no relationship between smoking and OA [86,87]. Direct mechanical joint injury also can cause acute inflammation, increase the production of IL-1β and IL-6 and chronic post-traumatic OA, low mobility and CVD [88,89].

In summary, there are several common risk factors between OA and CVD, including age, obesity and reduced physical activity, which directly or indirectly influence the progression of these diseases. Therefore, adequate physical activity, controlling appetite and regulation of food consumption and/or calorie restriction could help to minimize the risk for OA and CVD development [90].

### 2.2. Oxidative Stress and Other Common Molecular Mechanisms in CVD and OA

There are several theories suggesting a possible connection between OA and CVD. It is known that arthralgia, caused by OA, results in physical inactivity and can worsen CVD. In hypertension, the natural balance between vascular regulators, produced by endothelium, is disrupted, leading to the changes of vascular tone [91]. Hypertension patients show decreased peripheral blood flow and reduced consumption of nutrition and oxygen in myocardium, causing ischemia and cell death. The reduced blood flow also negatively affects subchondral bone, whose vessels nourish cartilage [92,93,94].

Oxidative stress is also an important feature of OA and CVD, and there are many reactive oxygen species released and activated in both clinical scenarios [95,96]. Activity of superoxide dismutase (SOD), catalase (CAT) and glutathione peroxidase was lower in patients with hypertension, which showed a decreased ability to overcome oxidative stress [97]. Thiobarbituric acid (TBARS), a side product of lipid peroxidation, was also increased in elderly patients with hypertension, whereas NO production was lower than in healthy individuals [97]. OA patients also had increased levels of oxidative stress marker malondialdehyde (MDA) in synovial fluid and decreased activity of SOD, CAT and glutathione S-transferase (GST) in blood serum [98,99]. 

The imbalance between the generation and elimination of reactive oxygen species leads to the activation of redox-sensitive signalling pathways, e.g., mitogen-activated stress kinase (MAPK) c-Jun N-terminal kinase (JNK), causing production of pro-inflammatory cytokines and the formation of inflammation. The inhibition of MAPK/JNK had a beneficial effect on both OA and CVD [100]. In addition, there is in vitro evidence that the inhibition of another mitogen-activated stress kinase p38 signalling pathway suppressed apoptosis and inflammation in human chondrocytes isolated from OA cartilage [101]. The inhibition of the JNK signalling pathway reduced the production of matrix metalloproteinases (MMP3 and MMP13) in mouse and human chondrocytes [102]. An in vivo study showed that the suppression of stress MAPK, JNK and p38 resulted in improved ventricular remodelling in spontaneously hypertensive rats [103].

OA and CVD also share some low-grade inflammatory mediators associated with increased risk, and major adverse cardiovascular events are also biomarkers of knee OA, e.g., cytokines interleukin 6 (IL-6) [104,105], interleukin 17 (IL-17) [106,107] and adipokines (leptin, adiponectin and resistin) [108,109].

In addition, an increasing amount of evidence suggests a significant association between symptomatic OA and incident coronary heart disease (myocardial infarction/coronary insufficiency syndrome), especially in patients with hand, knee and hip OA causing severe disability [110,111,112].

## 3. Ion Channel Regulators for the Treatment of CVD

There are few targets for CVD treatment: drugs that target ion channels and pharmacological agents that target the renin angiotensin-aldosterone (RAAS) system and adrenergic system. In this review, we focus on CVD drug targets that are ion channels and receptors for the sake of maintaining focus and producing a concise article. 

Arrhythmia is a very common heart disease, which is diagnosed when patient suffers an irregular heartbeat, causing the risk of collapse or sudden death. Therefore, in many cases, arrhythmias need long-term medication treatment, the majority of which are modulators of ion channels [113,114]. These drugs include amiodarone, flecainide and verapamil [115]. Arrhythmia and hypertension are diseases closely related to myocardial ischemia; therefore, the same drugs are often involved in their treatment strategies. They include ion channel blockers, and some of them are used in combination to treat CVD [116]. Myocardial ischemia can be reduced by β-adrenoblockers (e.g., propranolol, carvedilol) that are also prescribed in hypertension and heart failure for the improvement of final cardiovascular outcomes [117,118]. Potassium channel agonists (nicorandil, minoxidil) can be also used in chest angina, i.e., they open the adenosine triphosphate (ATP) sensitive K^+^ channel (KATP) or high-conductance calcium dependent K^+^ channel (BKCa) that change the polarization of the cell membrane [119,120]. In 2005, a new, more specific heart rate lowering agent ivabradine was registered by the European Medicines Agency (EMA). Ivabradine inhibits the so-called “funny current” (If) in the sinoatrial node [121]. The other new anti-ischemic drug, ranolazine, inhibits the late sodium current (INa) and causes rapid depolarization and activation of voltage-gated L-type calcium channels [122]. 

Cardiovascular drugs are systemically administered for long periods of time and therefore not only may affect myocardium or smooth vascular muscles but also can interact with ion channels on the cells of other organs, including subchondral bone, synovium and articular cartilage. 

## 4. Cardiovascular Drugs Directly and Indirectly Regulating Ion Channels

Taking into account the possible influence of CVD drugs on chondrocyte channelome, it is important to determine whether CVD drugs can modulate degenerative processes in OA joints. Both anabolic and catabolic responses of chondrocytes are well regulated by various types of ion channels, pumps, transporters and receptors [29]. Therefore, we have summarized the potential direct and indirect effects of cardiovascular drugs with the ion channel modulatory activity on chondrogenesis and cartilage degeneration (Table 1). We have found no data yet about the potential effect of approved CVD acting through potassium channels on the cartilage tissue or course of OA; therefore, this information is absent in Table 1. 

### 4.1. Inhibitors of Voltage-Gated Calcium (Ca^2+^) Channels in Chondrogenesis

**Figure 2:**
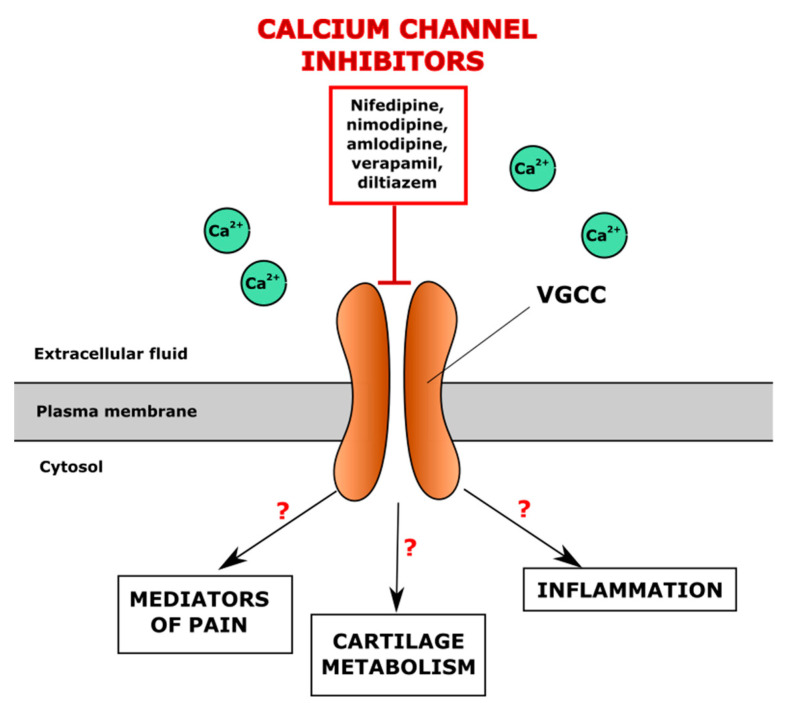
Potential regulation of iCa^2+^ levels in the chondrocyte through the direct blocking of voltage-gated calcium channels (VGCC) by antihypertensive drugs, VGCC inhibitors.

Voltage-gated ion channels represent a family of transmembrane proteins that play important roles in the electrical signalling in cells. The activity of voltage-gated ion channels is regulated by the cell membrane potential and opening channels, which allows the movement of ions along an electrochemical gradient across the cellular membranes. Voltage-gated ion channels are a class of transmembrane proteins that participate in the ion shuttling through the membrane and are activated by changes in the electrical membrane potential near the channel [143]. Voltage-gated calcium channels (VGCC) regulate entry of Ca^2+^, one of the most important second messengers, to the cell, initiating many different cellular events, including the depolarization and repolarization required for efficient electromechanical coupling in cardiomyocytes, regulation of enzyme activity, synaptic transmission, gene transcription, secretion, etc. [144]. Inhibitors of VGCC are known as one of the biggest groups of anti-hypertensive drugs. The pathogenesis of OA and CVD seems to have a common but neglected link through the altered intracellular Ca^2+^ (iCa^2+^) signalling, which may contribute to the development of comorbidities in some individuals. Since the L-type calcium channel Cav1.2 is abundant in chondrocytes [145], the inhibitors of VGCC channels, such as nifedipine, amlodipine, verapamil and diltiazem, commonly used for the treatment of CVD, may affect the articular cartilage by modulating several responses, including pain modulation, altered cartilage metabolism and reduction of inflammation; however, the exact mechanisms are not fully elucidated so far (Figure 2). The VGCC function in chondrocytes and pathogenesis of OA has been previously reviewed [146]. The possible effects of L-type calcium channel inhibitors on cartilage and regulation of OA development are summarised in Table 1. 

Several studies suggest that calcium channel (especially L-type Ca^2+^ channel) antagonists, such as verapamil and amlodipine, may have beneficial effects for patients by attenuating the progression of OA, increasing production of ECM components and reducing inflammation [123,147]. The lockage of the L-type Ca^2+^ channel by verapamil may not only improve the heart beat rate and correct blood pressure, but it also may have a chondroprotective effect and slow down the development of OA by supressing Wnt/β-catenin signalling. These mechanisms results in the inhibition of chondrocyte hypertrophy and the upregulation of the chondrogenic markers aggrecan, collagen II and transcription factor SOX9 [123,125]. On the contrary, an in vivo pilot study showed a negative effect of VGCC inhibitors (verapamil, nifedipine) in OA patients, as the drugs increased pain and discomfort [124]. 

Another active regulator of iCa^2+^ level is sarco/endoplasmic reticulum Ca^2+^-ATPase (SERCA). SERCA reduces the intracellular amount of calcium by pumping it back to the endoplasmic reticulum. The calcium regulator SERCA has several isoforms, while SERCA2 is the most widely expressed in different types of tissues [148]. In vitro studies showed the possible effect of SERCA inhibitors on cartilage. The inhibition of SERCA in chick embryo chondrocytes suppressed the expression of collagen II and proteoglycans (but not glycosaminoglycans) [149]. However, an increased expression of SERCA also increases MMP-13 in the C28/I2 chondrocyte cell line [150]. However, the studies performed so far are not sufficient to elucidate the involvement of SERCA regulators in OA.

### 4.2. Voltage-Gated Sodium (Na^+^) Channels in Chondrogenesis

The expression of five different voltage-gated sodium channels (VGSC) was detected in human cartilage, including Nav1.1β, Nav1.2β, Nav1.2α Nav1.3α and Nav1.6α [151]. VGSCs were shown to participate in maintaining the membrane potential of rabbit articular chondrocytes [152]. However, more detailed information about the effect of VGSC on the polarisation of the human chondrocyte membrane is still missing. VGSC inhibitors lidocaine and procainamide are not only anti-arrhythmic drugs but also local anaesthetics. Lidocaine is widely used in OA pain management, but an in vitro study showed a possible cytotoxic effect in bovine articular chondrocytes [127,153]. Thus, since in cardiology, lidocaine is used intravenously only for a short period, its long-term effect on OA joints is not clear yet [154]. However, high doses of lidocaine seem to have a toxic effect on the cartilage [127,153]. In addition, procainamide showed an increased amount of cartilage elements and Sox9 proteins in a zebrafish embryo model [155].

### 4.3. Potassium (K^+^) Channels in CVD and Chondrocytes Metabolism

The potassium channel superfamily is one of the most widely expressed families of membrane ion channels in human tissues. Potassium ions are involved in the regulation of chondrocyte volume and the proliferation and production of ECM [156]. Several types of potassium channels were identified in chondrocytes, including ATP-sensitive (K_ATP_), voltage-gated potassium channels (VGPC), calcium-activated potassium channels (K_Ca_), inward rectifier potassium channels (Kir), potassium channels and tandem pore potassium channels (K2P) [157]. So far, only the K_ATP_ channel opener (anti-anginal drug nicorandil) is used to treat CVD, particularly arrhythmias and chest angina [158,159]. The next section will explore this in more detail. 

**Figure 3:**
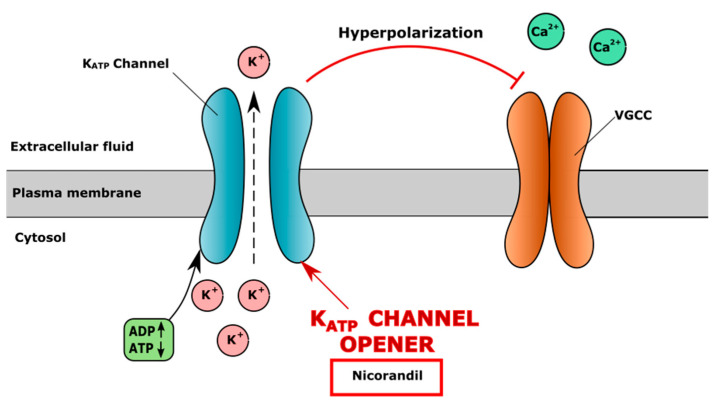
Mechanism of action of ATP-sensitive potassium (K_ATP_) channel opener nicorandil. Nicorandil opens K_ATP_ channel, causing extracellular efflux of K^+^, resulting in hyperpolarization of cell membrane and closure of voltage-gated calcium channel (VGCC), which results in decreased level of iCa^2+^.

K_ATP_ channels are activated by adenosine diphosphate (ADP), which, binding to the channel, activates the efflux of the potassium ion to the extracellular space, causing the hyperpolarization of the cell membrane and the inhibition of VGCC. In contrast, ATP blocks K_ATP_ channels, causing membrane depolarisation, the opening of VGCC and calcium flow into the cell [160]. An increase in ATP level was observed in a human chondrocyte culture after mechanical stimulation, which may lead to the inhibition of K_ATP_ channels and the opening of VGCC [161]. The K_ATP_ channel opener nicorandil stimulates membrane hyperpolarization, leading to the inhibition of the VGCC channel and a decreased intracellular level of Ca2+ (Figure 3).

Voltage-gated potassium channels (VGPC) were shown to drive changes of cell membrane potential and cause rapid Ca^2+^ oscillations [162]. Selective blockers of VGPC are toxins (e.g., scorpion venom) or small molecules (e.g., tetrabutylammonium) [163,164]. VGPC blockers (tetraethylammonium, 4-aminopyridine and a-dendrotoxin) were shown to cause the depolarisation of the cell plasma membrane and the inhibited proliferation of rat articular chondrocytes and expression of KV1.6, KV2.1, KV3.3 and KV4.1, which are found in cartilage [162,165]. Most VGPC inhibitors used in cardiology have multiple mechanisms of action, i.e., amiodarone, an antiarrhythmic medication, has a strong effect on metabolic functions due to the iodine group in the molecule, which binds to the thyroid receptors and possibly can cause hypothyroidism [166]. The VGPC inhibitors antiarrhythmic flecainide and quinidine can also suppress VGSC. 

**Figure 4:**
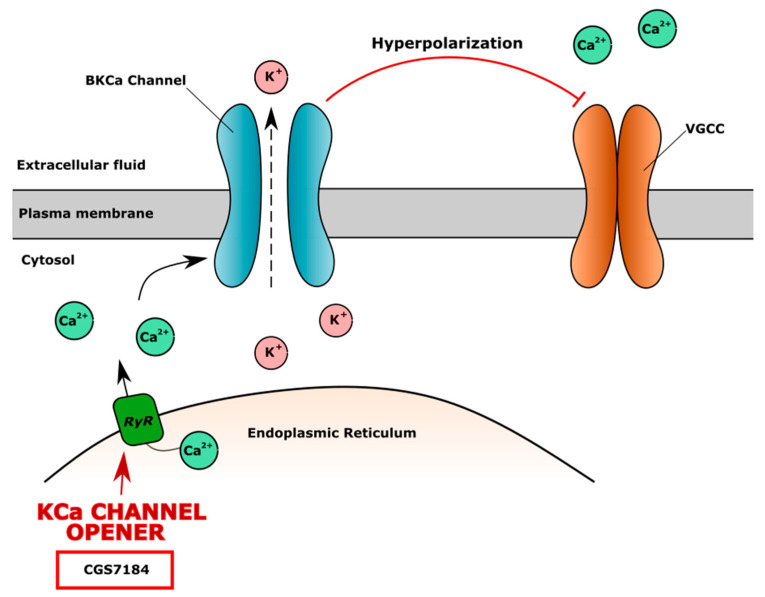
The mechanism of action of high-conductance calcium dependent big K^+^ channel (BKCa) opener CGS7184. CGS7184 opens ryanodine receptors (RyR) causing Ca^2+^ flow from endoplasmic reticulum to cell cytoplasm. Increased level of iCa^2+^ opens BKCa channels, causing efflux of K^+^, which leads to cell membrane hyperpolarization and inhibition of voltage-gated calcium channel (VGCC), resulting in lower level of iCa^2+^ and possible negative effect on cartilage.

High-conductance calcium-dependent big K^+^ channels (BKCa) are also widely expressed in chondrocytes. The opening of the BKCa channel causes potassium efflux into extracellular fluid and the hyperpolarization of the membrane. The increased extracellular level of K^+^ and membrane hyperpolarization blocks VGCC channels, leading to the lower level of iCa^2+^. The KCa channel agonist CGS7184 releases Ca^2+^ from the endoplasmic reticulum through ryanodine receptors (RyR), indirectly activates the BKCa channel and causes membrane hyperpolarization and closure of the VGCC channel (Figure 4). The ryanodine receptor isoform RYR2 is the major cellular mediator of calcium-induced calcium release (CICR) in mammalian cells [167]. 

It is known that the hyperpolarization of the cell membrane caused by osmolytes is related to many negative effects on human chondrocytes—voltage-gated ion channels are closed, the volume of the cells reduces and extracellular matrix production decreases [166,168]. BKCa activators are not yet used in cardiology, while several studies demonstrated their cardioprotective effect via the improvement of myocardial function and coronary flow [169,170]. A new KCa channel activator CGS7184 was shown to open the RyR2 channel in rat cardiac sarcoplasmic reticulum vesicles and increased the level of iCa^2+^, improving heart functioning [171]. 

### 4.4. Hyperpolarization-Activated Cyclic Nucleotide-Gated (HCN) Channels

**Figure 5:**
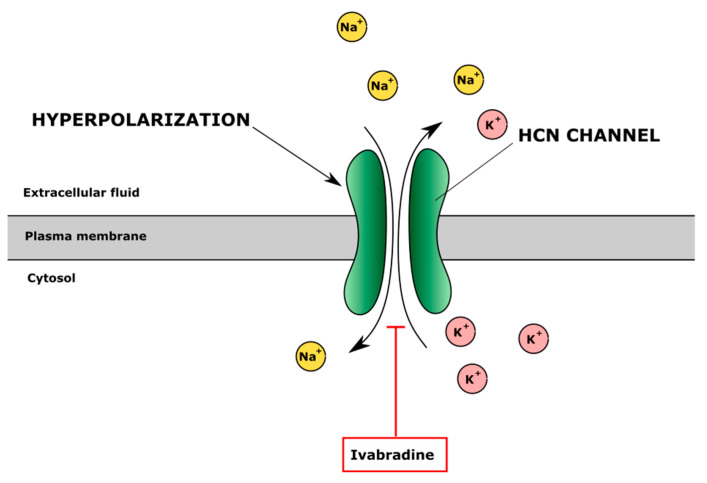
The effect of inhibitor of hyperpolarization-activated cyclic nucleotide-gated (HCN) channels ivabradine on ion regulation. Ivabradine blocks HCN channel inhibiting Na^+^ influx and K^+^ efflux and changes intracellular and extracellular ion balance and cell membrane potential.

Hyperpolarization-activated cyclic nucleotide-gated (HCN) channels regulate the permeability of Na^+^ and K^+^ ions through the cell membranes [172]. Ivabradine inhibits HCN channels and decreases K^+^ flow to extracellular space, which changes membrane potential and shortens the depolarisation phase in the myocyte membrane, causing bradycardia (Figure 5). Protective effects of ivabradine on TNF-α-treated primary human chondrocytes were observed [130]. Ivabradine also restored SERCA activity after myocardial infarction in rats [173]. The authors also showed that ivabradine inhibited the activation of NF-κB, which was elevated in patients with OA and had analgesic, anti-inflammatory and antioxidant properties. In addition, ivabradine reduced inflammation caused by TNF-α in human primary chondrocytes by diminishing levels of IL-6 and IL-1β [130]. Thus, HCN inhibitors seem to have complex activity, which can positively affect human chondrocytes and cartilage.

### 4.5. Complex Activity of Ion Channel Regulators

Furthermore, a number of commonly used CVD drugs may affect several ion channels at the same time. For instance, amiodarone (anti-arrhythmic drug, VGPC channel blocker) can also block L-type calcium, potassium and sodium currents; prolong phase 3 of the cardiac action potential; and reduce pulse rate in humans [174,175,176]. It was also shown that ibutilide (anti-arrhythmic drug, VGPC blocker) inhibited the activity of hERG potassium channels and delayed inward rectifier potassium and L-type calcium channels in Guinea pig cardiomyocytes [177]. The other antiarrhythmic drugs flecainide and quinidine work mainly by blocking the Nav1.5 sodium channel [178,179]. In addition, flecainide can also inhibit a ryanodine receptor 2 (RyR2), a major regulator of sarcoplasmic release of stored calcium ions [180]. The inhibition of sarcoplasmic release of Ca^2+^ was shown to have a negative effect on proteoglycan synthesis in cartilage [149]. Quinidine can also block certain VGPCs in Xenopus laevis oocytes [181] and act as a muscarinic and alpha-1 receptor blocker [182]. The cell membrane depolarisation increases the iCa^2+^ level. Ivabradine, an antianginal drug and HCN channel inhibitor, was also shown to partially inhibit human Cav1.2 and Nav1.5 in a human embryonic kidney (HEK) cell line [183]. 

In summary, multiple studies have shown that most of the drugs used to treat CVD can have multiple mechanisms of action via several channels, and very often it is hard to evaluate a specific and/or single channel modulation effect. Therefore, CVD drugs are likely to have complex activities in OA, including the modulation of inflammation, pain and cartilage ECM turnover. However, the effects of an altered iCa^2+^ level in joint tissues have not been fully investigated. 

## 5. CVD Drugs Indirectly Regulating Ion Channels

There are many CVD drugs that indirectly change the level of intracellular ions by binding to the non-ion channels or receptors. We will further discuss two main groups of the drugs used in cardiology: β-adrenergic system inhibitors and angiotensin-aldosterone system modulators.

### 5.1. Non-Selective Inhibitors of β-Adrenergic System

**Figure 6:**
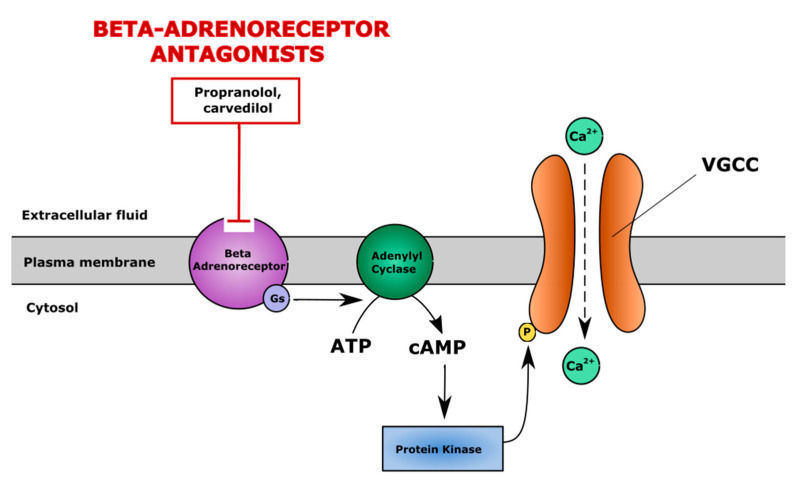
The mechanism of action of β-adrenergic system antagonists. β-adrenergic system antagonists (e.g., propranolol, carvedilol) block β-adrenoreceptors and suppress cAMP/PKA pathway-induced activation of voltage gated calcium channels (VGCC), maintaining a physiological level of iCa^2+^. cAMP—cyclic adenosine monophosphate, PKA—protein kinase A.

β-adrenergic receptors belong to the G-protein coupled receptor family. There are three main subtypes of β-adrenergic receptors: β-1 found in cardiac tissue, β-2 found in airway smooth muscle and β-3 found in adipose tissue [184]. The expression of β-2 adrenoreceptors (AdR) was also found in human and murine cartilage and in proliferating and hypertrophic chondrocytes [132,185,186,187]. The activation of β-adrenergic receptors is related to the regulation of iCa^2+^ in cardiomyocytes as well [188]. Endogenous catecholamines (epinephrine, norepinephrine) stimulate β-adrenergic receptors via G protein and activate adenylyl cyclase, causing the generation of cyclic adenosine monophosphate (cAMP). cAMP activates protein kinase A, which is able to phosphorylate L-type calcium channels and induce the influx of extracellular calcium into cytoplasm [188]. Inhibitors of the β-adrenergic system suppress the activation of the VGCC channel and prevent the increase of iCa^2+^ [189], which may alter the functioning of cartilage (Figure 6). 

Isoproterenol, a β-adrenergic system agonist, was shown to increase iCa^2+^ levels in rat cardiomyocytes and Jurkat T cells via the activation of RyR, causing calcium sparks from endoplasmic reticulum into the cytoplasm and resulting in increased heart contractility [190,191]. Carvedilol, a non-selective β adrenoreceptor blocker, upregulated SERCA2 expression which resulted in a decreased level of Ca2+ in rat cardiac ventricular myocytes [192]. β-2 adrenoreceptors also play a role in chondrogenesis, as their action via the MAPK ERK1/2 cascade was shown in vitro [186]. The activation of β-2 adrenoreceptors by isoproterenol downregulated the expression of collagen type II, pre-hypertrophic and hypertrophic gene collagen type X and Indian hedgehog (Ihh), respectively, and reduced matrix proteoglycan synthesis in prechondrogenic ATDC-5 cells and chondrocytes, isolated from the ribs of embryonic E18.5 mice [132,185,186]. Carvedilol restored IL-1β suppressed expression of aggrecan and Col II at protein levels in SW1353 human chondrocytes [134].

The regulation of the adrenergic system also affects subchondral bone. Blocking the adrenergic system by propranolol suppressed subchondral bone loss, but it had no effect on cartilage thickness, while isoproterenol intensified subchondral bone loss and caused cartilage degradation and proteoglycan loss in a rat temporomandibular joint osteoarthritis model [131]. Anti-inflammatory effects of adrenergic system modulators have been also demonstrated in human chondrocytes, isolated from the cartilage of OA patients. Norepinephrine, a non-selective stimulator of adrenoreceptors, blocked the pro-inflammatory effect of IL-1β, suppressed the production of IL-8 and MMP-13 and increased the expression of collagen type II and GAGs in human chondrocytes obtained from the articular cartilage of OA patients [193]. Thus, the stimulation of the adrenergic system has mostly negative effects on cartilage, suggesting that blockers of adrenoreceptors could be beneficial.

### 5.2. Modulators of Renin-Angiotensin-Aldosterone System (RAAS)

RAAS is one of the main targets in the treatment of CVD; however, such treatment can also affect cartilage structure and functions in few ways. First of all, components of RAAS (aldosterone) play a crucial role in blood vessels, including those of subchondral bone and synovial capillaries, by causing vascular inflammation or endothelium dysfunction [194,195,196]. Angiotensin II, a member of RAAS, can also activate the WNT-β-catenin pathway [197]. WNT signalling is extremely important for vascular development in embryos [198], as blocking the canonical WNT pathway inhibited the formation of vascular structures during embryonic stem cell differentiation in vitro [199]. Abnormal WNT signalling was observed in both cardiovascular pathologies and OA [200,201]. Losartan, an angiotensin II receptor inhibitor, also blocks the TGF-β1 signalling pathway [139], which plays a critical role in chondrogenesis, although it promotes fibrocartilage formation instead of hyaline-like cartilage [141]. Angiotensin converting enzymes I and II (ACE) also share some structural similarities with enzymes of the metalloproteinase family [202]. ACEs were found in the zone of cartilage maturation and are expressed by osteoblasts and hypertrophic chondrocytes in a murine femur fracture model [203], suggesting their involvement in cartilage development and regeneration. The ACE inhibitors captopril and enalapril also inhibited the conversion of procollagen to collagen in chick embryo cartilage cultures by blocking procollagen proteinases [119]. Moreover, the angiotensin receptor II type 1 was found on the surface of human articular chondrocytes [200], while the activation of this receptor suppressed hypertrophic differentiation of the ATDC5 chondrogenic mice cell line [201]. In vivo studies showed a range of effects of RAAS modulators on animal models. Captopril seems to have a positive effect on joint tissue structure by thickening cartilage, decreasing the hypertrophic zone and increasing the proliferative zone in a rat OA model [136], elevating subchondral trabecular bone volume and density in DOCA hypertensive rats [138]. The application of losartan showed contradictory results, as it promoted chondrocyte hypertrophy during mice skeletal development [142], whereas it ameliorated histological markers of OA in a murine OA model with chondrodysplasia gene mutation [139] and promoted cartilage healing in a rabbit osteochondral model [140,141]

The RAAS can also modulate calcium signalling in the Chinese hamster ovary (CHO) cell line, as the ACE generated calcium signalling in the cells via the inositol 1,4,5-triphosphate (IP3) signalling pathway [204]. In addition, the ACE inhibitor enalapril and angiotensin receptor II blocker losartan stimulated the expression and function of SERCA2a/b in the left ventricle of rats after myocardial infarction [205]. Similarly, in chondrocytes, by changing calcium signalling, RAAS may also potentially affect iCa^2+^ level and all processes that are regulated by calcium.

Thus, an abundant number of recent studies showed that the blockers of angiotensin-aldosterone and β-adrenoreceptor systems, in addition to beneficial effects on hypertension in humans, may modulate the course of OA.

## 6. Conclusions

The variety of ion channel inhibitors, abundantly and protractedly used to treat CVD, are likely to modulate ion channel-mediated events in chondrocytes, which in turn affects their metabolism, osmotic responses, turnover of cartilage ECM and inflammatory responses. The HCN channel inhibitor ivabradine had cartilage-ECM production stimulating responses in vitro [130]. Members of the β-adrenoreceptor inhibitors group may diminish adrenergic system-related adverse effects on chondrogenesis, such as reduced ECM production [186], while angiotensin-aldosterone system modulators (captopril, enalapril, losartan) showed both stimulatory and inhibitory effects in cartilage regeneration.

Direct VGCC calcium channel inhibitors (verapamil, amlodipine, lercanidipine) also are likely to have complex beneficial activities in OA, including anti-inflammatory properties, modulation of chondrogenesis and pain management [123]. However, there is no consensus on the effects of VGCC inhibitors on human cartilage, which might be related to the rapid compensation of iCa^2+^ levels from intracellular stores (i.e., sarco/endoplasmic reticulum) [206]. Drugs regulating cell membrane hyperpolarisation-induced inhibition of VGCC, as well as inhibition of SERCA channels, may have weighty effects on the function of chondrocytes. The structure of subchondral bone is also likely to be affected by cardiovascular drugs; however, too scarce information is available in this field to draw more solid conclusions. Further studies are needed to document complex clinical outcomes of CVD drugs, revealing their aggravating or beneficial effects on the development and progression of metabolic, mechanical load-related or inflammatory OA phenotypes.

Currently, there are no reliable, quantifiable and easily measured biochemical markers capable of providing an earlier diagnosis of OA, informing on the prognosis of OA disease and monitoring the responses to emerging therapeutic modalities [25]. The evaluation of structural changes in articular damage via imaging biomarkers (as determined by radiograph or magnetic resonance imaging (MRI)) is the most frequently used method in clinical trials to evaluate subject eligibility and/or the efficacy of interventions, by ascertaining treatment effects on joint structures. The implication of CVD drugs in the aetiopathogenesis of OA should be considered when prescribing ion channel modulators, particularly in long-term therapy protocols.

## Figures and Tables

**Table 1:** Potential effects of the direct and indirect ion channel modulators used in treatment of CVD on the development of OA.

Drug	Role in CVD	Effect on Cartilage In Vitro	Effect on Cartilage in Animal Models In Vivo	Effect on Patients	References
Direct ion channel modulators					
Voltage-Gated Calcium Channel Blockers					
Verapamil	Inhibits VGCC in vascular smooth muscle and myocardial tissue, reduces peripheral vascular resistance and heart contractility. Indications: hypertension, chest angina, arrhythmia	Inhibition of chondrocyte proliferation, decreased number of hypertrophic chondrocytes, upregulation of chondrogenic markers (ACAN, COL2A1, SOX9), downregulation of AXIN2 and MMP3 in human osteoarthritic chondrocytes	-	Worsened OA patients’ condition based on Lequesne scoring system	[123,124,125]
Nifedipine	Inhibits VGCC in vascular smooth muscle cells, causing smooth muscle relaxation and decrease of blood pressure. Indications: hypertension, chest angina	Inhibition of chondrocyte maturation and proliferation in bone marrow-mesenchymal stem cells and chondrocytes; upregulated production of GAGs and collagen type II in human chondrocytes	-	No effects in OA patients according to Lequesne scoring system	[124,126]
Amlodipine, lercanidipine, felodipine, nitrendipine		-	-	Improved Lequesne index in OA patients	[124]
Voltage-Gated Sodium Channel Blockers					
Lidocaine	Inhibits VGSC, decreases the depolarization, automaticity and excitability in the ventricles Indication: arrhythmia	Chondrotoxic effect; necrosis in equine and bovine articular chondrocytes	-	Reduced pain in OA patients	[127,128,129]
Hyperpolarization-Activated Cyclic Nucleotide-Gated Channel Inhibitors					
Ivabradine	Lowers heart rate by selectively inhibiting If channels (“funny channels”) in the heart and prolonging diastolic depolarization Indication: heart failure	Reduced expression of matrix metalloproteinases (MMP-3 and MMP-13), ADAMTS-4 and ADAMTS-5 in primary human chondrocytes	-	-	[130]
Indirect ion channel modulators					
β-adrenoreceptor Inhibitors					
Propranolol	Nonselective β-adrenergic receptor antagonist Indications: hypertension, angina, arrhythmia	Promoted chondrogenic differentiation to hypertrophic chondrocytes by increasing Col I and Col X gene expression, decreasing SOX6 expression in murine pre-chondrogenic ATDC5 cells; suppressed subchondral bone loss in rat model.	-	-	[131,132]
Carvedilol		Reversed IL-1β induced downregulation of aggrecan and Col II protein in murine pre-chondrogenic ATDC5 cells in SW1353 chondrocytes	-	-	[133,134]
Angiotensin-Aldosterone System Modulators					
Captopril	Inhibitor of angiotensin-converting enzyme Indications: hypertension, heart failure	Inhibited reversion of procollagen to collagen in cartilage and tendon cell culture	Increased thickness of articular cartilage, decreased hypertrophic zone and increased proliferative zone in rat OA model	-	[135,136,137,138]
Enalapril		Inhibited reversion of procollagen to collagen in cartilage and tendon cell culture	-	-	[135]
Losartan	Angiotensin II receptor inhibitor Indications: hypertension, heart failure	-	Increased OA progression according to histopathological scoring in murine OA model; increased Col10a1 expression in mice; diminished degradation of cartilage in mice; enhanced hyaline-like rabbit cartilage healing	-	[139,140,141,142]
